# Local environmental factors drive distributions of ecologically-contrasting mosquito species (Diptera: Culicidae)

**DOI:** 10.1038/s41598-024-64948-y

**Published:** 2024-08-20

**Authors:** Roel M. Wouters, Wouter Beukema, Maarten Schrama, Koos Biesmeijer, Marieta A. H. Braks, Pepijn Helleman, Francis Schaffner, Joey van Slobbe, Arjan Stroo, Jordy G. van der Beek

**Affiliations:** 1 NL Biodiversity and Society Research Group, Naturalis Biodiversity Center, 2333 CR Leiden, The Netherlands; 2 Institute of Environmental Sciences, Leiden University, 2333 CC Leiden, The Netherlands; 3 Department of Ecology, Faculty of Science, Charles University, 12844 Prague, Czechia; 4 RAVON, Reptile, Amphibian and Fish Conservation Netherlands, 6501 BK Nijmegen, The Netherlands; 5 Centre for Zoonoses and Environmental Microbiology, Centre for Infectious Disease Control, National Institute for Public Health and the Environment, 3721 MA Bilthoven, The Netherlands; 6 Francis Schaffner Consultancy, 4125 Riehen, Switzerland; 7 Bonaire Public Health Department, Public Body Bonaire, 4PXG+GH4, Kralendijk, Dutch Caribbean The Netherlands; 8 Centre for Monitoring of Vectors (CMV), Netherlands Food and Consumer Product Safety Authority (NVWA), 6706 EA Wageningen, The Netherlands; 9 Pandemic and Disaster Preparedness Center, Delft, Rotterdam, The Netherlands

**Keywords:** Ecology, Biodiversity, Biogeography, Ecological modelling, Invasive species, Tropical ecology

## Abstract

Mosquitoes are important vectors of disease pathogens and multiple species are undergoing geographical shifts due to global changes. As such, there is a growing need for accurate distribution predictions. Ecological niche modelling (ENM) is an effective tool to assess mosquito distribution patterns and link these to underlying environmental preferences. Typically, macroclimatic variables are used as primary predictors of mosquito distributions. However, they likely undervalue local conditions and intraspecific variation in environmental preferences. This is problematic, as mosquito control takes place at the local scale. Utilising high-resolution (10 × 10 m) Maxent ENMs on the island of Bonaire as model system, we explore the influence of local environmental variables on mosquito distributions. Our results show a distinct set of environmental variables shape distribution patterns across ecologically-distinct species, with urban variables strongly associated with introduced species like *Aedes* *aegypti* and *Culex* *quinquefasciatus*, while native species show habitat preferences for either mangroves, forests, or ephemeral water habitats. These findings underscore the importance of distinct local environmental factors in shaping distributions of different mosquitoes, even on a small island. As such, these findings warrant further studies aimed at predicting high-resolution mosquito distributions, opening avenues for preventative management of vector-borne disease risks amidst ongoing global change and ecosystem degradation.

## Introduction

Mosquitoes belong to the family of Culicidae with approximately 3700 species^1^. A subset of these species plays an important role in global public health as vector of infectious diseases for humans, livestock and wildlife. According to recent estimations, by 2050, half of the world’s population will be at risk of vector-borne diseases^2^. As a result of global change, many mosquito species are on the move^3–6^. Hence, there is a growing need to accurately predict mosquito distributions. As such, it is essential to understand the underlying drivers of mosquito distributions.

Large scale spatial projections of mosquito distributions rely on the predictive value of large-scale climatic factors (i.e. temperature and precipitation), resulting in relatively coarse-scale maps of species distributions^2,4,7–11^. However, at the local scale (< 10 km^2^), these climatic factors are largely indistinctive and local scale environmental parameters are thought to dictate the heterogeneity in mosquito distribution and local distribution maps of mosquitoes are generally lacking^12–16^. This greatly hampers the use of distribution maps to guide policy, e.g., to guide vector-control and other management/mitigation strategies that operate at similar local levels^17^. Hence, there is a growing need to include (local) environmental conditions, other than climatic variables in understanding mosquito distributions^12,13,17^.

Species distribution modelling, also known as ecological niche modelling (ENM), allows for fine-scale predictions of species occurrences^18–20^. ENMs comprise a family of statistical or machine-learning models, which may be used to predict the potential distribution of a given species in a given area by relating occurrence data to ecologically-relevant environmental conditions^21^. While ENMs have previously been used to generate global distribution maps largely relying on abovementioned climatic variables^2,4,7–11^, the models also allow for the inclusion of more local-scale environmental variables thus contributing to the understanding of the fine scale distribution of a given species. As such, ENM holds the potential to be applied for vector-borne disease management efforts, improving traditional disease risk maps and increasing knowledge on the ecology of a given species^18^.

Indeed, studies applying fine-scale ENM highlight the potential importance of local-scale variables^12,17^. For example, the occurrence of the (introduced) arbovirus vectors *Aedes* *aegypti* and *Aedes* *albopictus* in a human-dominated landscape in the tropics and subtropics can be explained by a number of key urban and nature-related factors (including population density, house density and distance from vegetation or water), and to a lesser extent also by local climatic factors such as temperature and precipitation^20,22–25^. However, there is a major bias in ENM-based studies on mosquitoes: the vast majority is geared towards a limited number of species involved in disease transmission, while preciously little is known about the distribution of other species^26^. This greatly limits our understanding of the importance of environmental variables in shaping the distribution of ecologically divergent mosquito species and in particular how introduced invasive species differ from the established native species.

**Figure 1:**
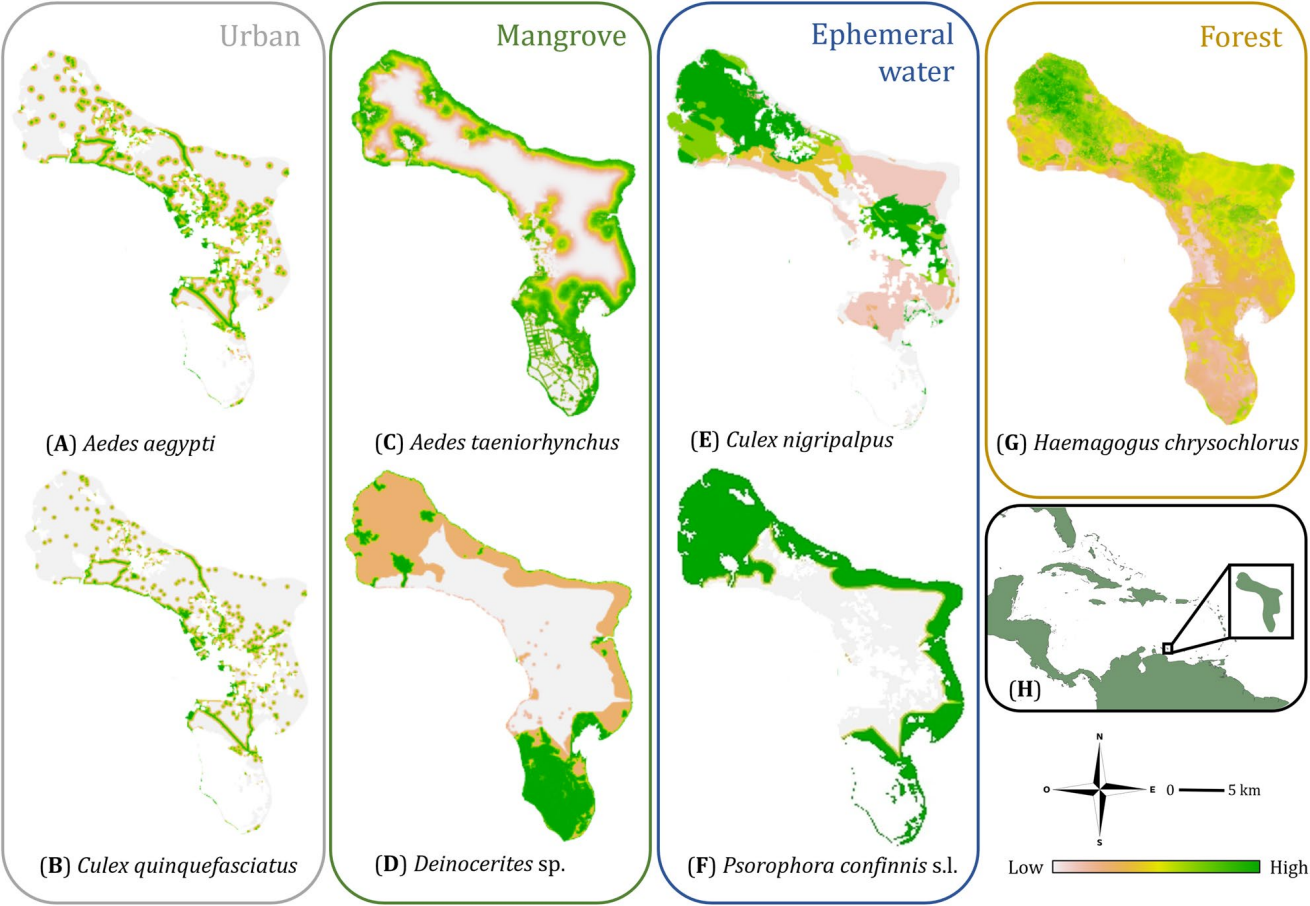
Habitat suitability maps for the seven modelled mosquito species: (**A**) *Aedes* *aegypti*, (**B**) *Culex* *quinquefasciatus*, (**C**) *Aedes* *taeniorhynchus*, (**D**) *Deinocerites* sp., (**E**) *Culex* *nigripalpus*, (**F**) *Psorophora* *confinnis* s.l., (**G**) *Haemagogus* *chrysochlorus*. (**H**) A map of the island of Bonaire as part of the Lesser Antilles, located near coast of Venezuela. Habitat suitability (relative likelihood) is illustrated with a gradient fill, where brown indicates low habitat suitability and green indicates high suitability. Species are grouped by corresponding habitat type.

Here, we use the island of Bonaire (288 km^2^), one of the islands of the Lesser Antilles in the Caribbean region (Fig. 1H), as a model to show how a confined set of key environmental factors at high resolution can be used to explain the local distributions of mosquito species with contrasting ecological strategies.

## Results and discussion

Habitat suitability (relative likelihood) for seven out of twelve mosquito species collected on Bonaire was estimated using a high-resolution ENM approach involving variable selection procedures, null model validation, and model selection to obtain an optimally parameterised model for each species. For five out of the twelve species, models could not be generated because the number of observations was insufficient to run the models (see Fig. S1 and Table S1). The most important contributing environmental variables for each species and the model performance (i.e., AUC and Boyce index) varied between mosquito species (Table 1), resulting in strikingly different predicted distribution patterns of the species on the island (Fig. 1). Below, the key findings concerning overall model performance and the important environmental factors contributing to the habitat suitability for the seven species are reported. See Table S2 and Fig. S2–S8 for complete model selection, performance and variable response curves per species and see Table S3 for an overview of all spatial data included in the models.

**Table 1:** Variable importance (%), ecology known from the literature and model performance (AUC values) for each modelled species.

Species	Contributing environmental variables				Known ecology	AUC value	Boyce index
	Variable 1	Variable 2	Variable 3	Variable 4			
*Aedes* *aegypti*	*Distance* *to* urban habitat (100%)				Introduced; container breeder; urban^28,35^	0.65	0.41
*Culex* *quinquefasciatus*	*Distance* *to* urban habitat (79%)	*Land* *use* Mangrove habitat (21%)			Introduced; nutrient-rich, dirty standing water breeder; urban^28^	0.64	0.31
*Aedes* *taeniorhynchus*	*Distance* *to* mangrove habitat (58.4%)	*Distance* *to* natural habitat (18.8%)	Elevation—negative correlation (12.9%)	*Land* *use* mangrove habitat (9.8%)	Breeds in fresh, brackish and saline waterbodies; mangrove^27,28^	0.89	0.74
*Deinocerites* sp.	*Distance* *to* protected nature area (54.4%)	*Distance* *to* mangrove habitat (46%)			Land crab-hole breeder; brackish and saline water tolerant; restricted to land crab holes of the family Gecarcinidae^29,38^	0.78	0.47
*Culex* *nigripalpus*	*Distance* *to* temporary waterbody (84.5%)	*Distance* *to* urban vegetation (15.5%)			Breeds in flooded ditches and small ponds; related to rainfall and ephemeral waterbodies^27,28^	0.50	0.11
*Psorophora* *confinnis* s.l	*Distance* *to* protected nature area (85.1%)	*Distance* *to* temporary waterbody (14.9%)			Breeds in flooded plains and ponds; related to rainfall and ephemeral waterbodies^28,39^	0.56	0.63
*Haemagogus* *chrysochlorus*	*Distance* *to* natural habitat (48%)	Elevation—positive correlation (18.6%)	*Land* *use* forest and high scrub habitat (12.4%)	*Geology* high terrace (7.2%)	Tree-hole breeder^29,37^	0.56	− 0.11

Cumulative contribution of the variables is at least 80%. The *distance* *to* variables are all negative correlations where increasing distance means lower suitability.

### *Aedes aegypti *and *Culex quinquefasciatus*

Both *Ae.* *aegypti* and *Cx.* *quinquefasciatus* show a relatively similar predicted occurrence over the island (Fig. 1A,B). Models performed moderately (AUC = 0.65 and 0.64, and Boyce index = 0.41 and 0.31 respectively) and the most important variable contributing to the habitat suitability of both species is the distance to urban habitat (100% and 79% respectively, Table 1), which is negatively correlated thus implying that habitat suitability decreases with increasing distance to urbanisation. For *Cx.* *quinquefasciatus* also land use (mangrove habitat, 21%) contributes to the habitat suitability. The predicted habitat suitability maps for these two species (Fig. 1A,B) reveal a wide distribution over the island with urban environments including the cities of Kralendijk (central west) and Rincon (northwest), as well as the main roads and other urban elements as important predictors.

### *Aedes taeniorhynchus*

In the model for *Ae.* *taeniorhynchus*, the most important contributing environmental variable is the distance to mangrove habitat (58.4%). The negative correlation between this variable and the occurrence of this species implies that the likelihood of observing *Ae.* *taeniorhynchus* decreases further away from mangrove habitat. Distance to nature is the second important variable (18.8%). Elevation (negative correlation) and the categorical variable mangrove habitat contribute respectively 12.9% and 9.8% (Table 1). The selected model has a high performance with AUC = 0.89 and Boyce index = 0.74. The predicted habitat suitability for *Ae.* *taeniorhynchus* is particularly high at lower elevations, including the salt plains and mangrove habitats in the south (Fig. 1C).

### *Deinocerites* sp.

The model for the yet undescribed species of *Deinocerites* sp. performs well with an AUC of 0.78 and a Boyce index of 0.47. The most important variable contributing to the habitat suitability of *Deinocerites* sp. is the distance to protected natural area (54.4%). The second contributing variable is the distance to mangrove habitat (46%, Table 1). Both variables show negative correlations with the respective environmental variables, implying that the probability of occurrence decreases further away from these habitats. A very high predicted suitability was observed in the southern part of Bonaire, where the protected salt plains are located (Fig. 1D). The vegetation on these salt plains consists predominantly of (low shrub) mangrove, and the waters are brackish or (hyper) saline; this is also the location with highest observed densities of land crabs which are closely associated with this species. Several suitable hotspots can also be found in the north in protected mangrove habitats.

### *Culex nigripalpus*

The model for *Cx.* *nigripalpus* showed that the two most important contributing variables are (1) the distance to temporary waterbodies (84.5%) and (2) the distance to urban vegetation (15.5%), which includes city parks and other green areas within the urbanised areas (Table 1). Both variables show no clear correlation (see Fig. S7) and the model performance is poor with an AUC of 0.5 and a Boyce index of 0.11). The habitat suitability map predicts high suitability in the north and northwest (i.e. in a protected nature area, agricultural habitat and around the city of Rincon) and east of Kralendijk (Fig. 1E), both areas consisting primarily of agricultural and unprotected nature areas. Several suitable habitats can be found in the southeast, where protected mangrove vegetation is dominant.

### *Psorophora confinnis* s.l.

For *Ps.* *confinnis* s.l., 85.1% of the modelled habitat suitability can be explained by the distance to protected nature area and the remaining 14.9% is explained by the distance to temporary waterbodies (Table 1). The model predicts high habitat suitability for this species in the central north and central south, which are both protected nature areas, as well as along the east coast (Fig. 1F). The model has a poor to moderate performance with an AUC of 0.56 and a Boyce index of 0.63.

### *Haemagogus chrysochlorus*

*Haemagogus* *chrysochlorus* was found at 23 unique sampling locations, the largest sample size of all modelled species. Nevertheless, the selected model had a poor performance (AUC = 0.56 and Boyce index = − 0.11). The four most contributing environmental variables were respectively: (1) distance to natural habitat (48%), (2) elevation (18.6%), (3) land use (forest and high scrub habitat, 12.4%) and (4) geology (higher terrace, 7.2%) (Table 1). For *Hg.* *chrysochlorus*, the model predicts high suitability in the higher elevated areas which are covered with forests containing trees of low height. Those areas are located in the north and centre of the island, both in protected natural areas. Several smaller suitable spots on the map are located central east and central south (Fig. 1G).

### Model performance and validation

We observe the lowest AUC validation scores for *Cx.* *nigripalpus* and *Hg.* *chrysochlorus* (AUC of 0.50 and 0.56, and Boyce index of 0.11 and − 0.11 respectively). For *Ps.* *confinnis* s.l. we observe a low AUC score of 0.56 but a better Boyce index score of 0.63. As we applied random stratified sampling based on the available habitat types, we do not expect a collection bias is the cause of these low validation scores. We argue instead that lacking environmental information at local to micro scales affects model performances in these species. Specifically, information (in the form of GIS layers) in the form of ephemeral waterbodies and tree hole availability, which are used as breeding sites, is currently not available at spatial broad scales. Inclusion of such small-scale landscape elements could likely improve model performance in the future. These relatively poor model performances (Table 1) imply that these ENM outputs need to be interpreted with caution and further ecological validation is necessary to affirm model results. Nevertheless, the contributing environmental variables explaining their distribution are largely in line with the known ecology of these species throughout their geographic distributions^27–32^ (Table 1). This underscores the notion that, despite the suboptimal performance, these models may still be useful to generate good local projections of the occurrence of the species of interest.

In the sampling data set, larval, pupal and adult stages of the modelled species were grouped to ensure a large enough sample size for model input. However, adult mosquitoes have the ability to disperse in contrast to the strictly water-bound larvae and pupae. Adults of some species, notably *Ae.* *taeniorhynchus*, are known to be able to cover large distances^33,34^. Interestingly, our findings show a *distance* *to* variable has the highest explanatory contribution for all seven species, suggesting sample sizes were robust enough to account for the dispersal of adult stages. This stresses the value of including *distance* *to* matrices when modelling flying insects such as mosquitoes. Using data from only a single life stage could potentially underestimate (larval) or overestimate (adult) the potential distribution and spatial invasion risk.

### Distinct environmental variables shape distributions

There was a remarkable difference between the predicted distribution of the introduced species (*Ae.* *aegypti* and *Cx.* *quinquefasciatus*; most likely introduced on Bonaire in the fifteenth th/sixteenth century^35,36^) on the one hand and mosquitoes that are likely of native origin (*Ae.* *taeniorhynchus*, *Deinocerites* sp., *Cx.* *nigripalpus*, *Ps.* *confinnis* s.l. and *Hg.* *chrysochlorus*) on the other hand. The introduced mosquito species show a distribution strictly centred around urban environments which matches the known ecology and lifecycle of these species along their distribution globally and in the Caribbean region^2,28,35^. Inclusion of more high-resolution urban spatial layers, for instance dealing with urban structure or demography, might reveal more detailed environmental information about the local distribution of these species that play an important role in public health.

In marked contrast to the urban-dwelling species discussed above, the distribution of the various native mosquito species is explained by different elements of the natural environment. Based on the predicted habitat suitability outcomes of the models, their distribution appears to reflect three separate habitat preferences: (1) mangrove-dwelling species, (2) forest-dwelling species and (3) ephemeral water-dwelling species (Fig. 1). The models for the native mangrove-dwelling mosquitoes (i.e. *Ae.* *taeniorhynchus* and *Deinocerites*. sp.) show a distribution closely aligned with natural mangrove habitats, which are predominantly located on the coastal areas. This coincides with the known ecology and breeding habits of these salt tolerant, mangrove breeding species^27,28^. The habitat suitability maps for these species suggest possible co-occurrence which was confirmed in our field survey. Two other species show a clear habitat preference towards ephemeral fresh waterbodies restricted to natural areas (*Ps.* *confinnis* s.l.) or vegetation-rich areas in urban environments (*Cx.* *nigripalpus*). This matches previous observations as both species are known to depend on ephemeral fresh waterbodies like natural and artificial ponds and floodplains^27,28^. To further increase model performance for this specific group, more fine-scale spatial information on different kinds and stages of ephemeral waterbodies would likely be needed. The forest-dwelling species group is represented by a single species: *Hg.* *chrysochlorus*. This species typically breeds in tree holes^29,37^, a behaviour consistent with our observed field data and the predicted distribution in forests at slightly elevated elevations on the high terrace of Bonaire. Notably, this region has many older trees, primarily *Guaiacum* *officinale* L., which frequently harbour tree holes. Additionally, larvae of the species were recorded in rockpools with abundant leaflitter within the same area. Overall our predicted maps closely match the known ecology of the species in this study, thus highlighting the strength of this approach to generate fine-scale distribution maps for mosquito species. However, this strongly relies on the availability of information on the local ecological factors at a given place.

## Conclusion

The results of this study show that, on a small but ecologically diverse island used as a model system, contrasting distributions of mosquito species can be explained by a set of ecologically-relevant environmental variables using the ENM approach Maxent. We observed a clear distinction between introduced species, of which the distributions were primarily explained the distance to urbanisation, and the native mosquitoes, of which the distribution is primarily explained by environmental predictors that are characteristic of natural habitats. Within the native mosquito species pool, predicted distributions were associated with three distinct habitat associations: mangrove-dwelling species, forest-dwelling species, and ephemeral water-dwelling species, which is largely in line with the known ecology of the modelled species despite poor model performances for three of seven species. Overall, our data implies that high-resolution ENM modelling reveals crucial insights in local habitat preferences that may ultimately benefit vector management and control. As such it can be a highly valuable tool to generate predictions on local mosquito distributions, but this relies strongly on the availability of high resolution data on local environmental factors. With increasing availability of remote sensing techniques leading to broad-scale, high-resolution spatial data, the application of ENM approaches for modelling vectors and their relatives will likely become more important in predicting distributions. This paves the way for generating fine-scale distribution maps that may benefit local policy and management (e.g. vector-control strategies) and other management practice related to (wildlife) veterinary and public health.

## Methods

### Study site and mosquito diversity survey

The island of Bonaire, located in the Lesser Antilles, is part of the Dutch Caribbean and with an area of 288 km^2^, it is one of the smaller inhabited islands in the Caribbean region. The island is located 80 km off the coast of Venezuela and has a semi-arid tropical savanna climate. Bonaire is primarily covered by limestone with basalt lava rock formations breaking through on the higher elevations^40^. The vegetation mainly consists of low shrub cacti and acacias with some ruminant low forest on the higher elevations. Both in the south and the north, protected mangrove vegetation surrounds the numerous salt lakes, locally called saliñas^40^. We conducted a thorough mosquito diversity survey on the island of Bonaire in December 2022 as part of a larger inventory of the mosquito diversity on the Dutch ABC islands (Aruba, Bonaire and Curaçao). The survey was conducted for a period of fourteen days (from 30 November until 14 December 2022). We sampled during the end of the rainy season (September through December), which is generally a favourable time for mosquito activity due to the presence of different types of waterbodies. The rainy season of 2022 was incredibly wet, with almost double the amount of precipitation compared to the annual average^41^. We conducted random stratified sampling based on the diversity of habitat types present on Bonaire. We selected the different habitat types based on existing vegetation and land use maps^40,42^ (Table S3) and we sampled all (aquatic) habitat types in Bonaire (excluding deep sea and shallow coastal waters, as they do not form suitable habitat for mosquitoes in general). Habitats were reached either by car or by foot and places suitable for trapping were identified on sight and with expertise from local collaborators.

### Mosquito collection

Mosquitoes are holometabolic insects, meaning they undergo complete metamorphosis and therefore have a larval and pupal stage before reaching their imago (adult) stage. To get a complete overview of the diversity, the used sampling methods were aimed to trap all larval, pupal and adult stages. Larvae and pupae are strictly bound to water and therefore, they were sampled from waterbodies using traditional dipping methods^43^. Adult specimens were trapped using traps baited with CO_2_ produced by yeast (Biogents Pro trap^44^), as well as using aspirators and netting techniques. CO_2_-baited traps were placed for a period of 24 h, covering diurnal, nocturnal and crepuscular species. From each trapping site corresponding coordinates and habitat type were registered, along with other environmental parameters (including pH and salinity of the water). Coordinates and corresponding environmental information were later attributed to the identified species. Collected samples were morphologically identified on-site to species level using a compilation of identification keys^27–29,45–49^. We omitted specimens that could not be identified to species level from further modelling. Table S1 shows all species identified from Bonaire.

### Data preparation, modelling pipeline and model selection

Occurrence data cleaning, preparation of environmental variables used to characterise species distributions, and ENM building were conducted in the R (version 4.2.2) open source statistical programming language (R Core Team 2022). Packages used include *data.table* (v.1.14.8)^50^ and *dplyr* (v.1.1.2)^51^ (general functions), *ncdf4* (v.1.22)^52^*,* *raster* (v.3.6.26)^53^*,* *rgdal* (v.1.6.7)^54^*,* *rgeos* (v.0.6.4)^55^*,* *sf* (v.1.0.14)^56^*,* *sp* (v.2.1.2)^57^, and *vegan* (v.2.6.4)^58^ (spatial functions), and *ade4* (v.1.7.22)^59^*,* *dismo* (v.1.3.14)^60^*,* *ecospat* (v.3.5.1)^61^*,* *ENMeval* (v.2.0.4)^62^*,* *ENMTools* (v.1.0.6)^63^, and *spatialEco* (v.2.0.1)^64^ (ENM-related functions).

We exclusively used occurrence data of specimens we collected during our field survey and did not work with open databases or formerly collected data. See Fig. S1 for a spatial rendering of the occurrence data for all modelled species. For the occurrence data, no distinction was made between the life stages (i.e. larval, pupal and adult stages) and only one occurrence point per unique location where a given species was present was used. This means only presence was taken into account, not abundance. Species with less than four unique collection sites were not modelled. Subsequently, we prepared a variety of biotic and abiotic environmental spatial layers. We started with publicly available layers which included layers that are known to be important for explaining mosquito distributions (e.g. urban habitat). Spatial layers include land use, vegetation, geology, soil type, elevation, protected nature areas, information on riverbeds and larger semi-temporary waterbodies and urban variables including urban areas, neighbourhoods, roads and footpaths. We did not provide a priori environment-species interactions and therefore initially included all spatial layers in each species model (see Table S3 for all base maps used for the environmental spatial layers). Because of its small size, Bonaire has just one macroclimatic zone. Therefore, no macroclimatic or weather variables were added to the models. Notably, a recent study examining the impact of climate change on Bonaire, did not distinguish more than one macroclimatic region on the island in their modelling approaches^65^. No microclimatic variables (e.g. higher temperature or lower humidity because of differences in urbanisation and tree cover) were available for Bonaire, and as such these could not be included. We rescaled the spatial layers with an original resolution of 2 × 2 m or 5 × 5 m to a resolution of 10 × 10 m in R and QGIS and we changed the coordinate system to WGS84. For most of the categorical layers, we created a *distance* *to* matrix as well. This enables numerical analyses of these variables and the importance of the proximity to a specific category.

Our chosen presence-background ENM approach needs a dataset of ‘background sites’ that are compared with the environmental variation at locations where mosquitos were collected. Therefore, we created such a background dataset by randomly selecting a total of 10,000 occurrence points within the well-sampled areas to prevent overprediction of the model^62,66,67^. As a threshold, only background areas were selected when at least two or three species were collected.

The machine-learning ENM algorithm Maxent^66^ was used to generate suitability predictions via the R package *ENMeval*^62^. Maxent is one of the most widely used SDM algorithms, often outperforms other ENMs, and generally achieves good results when using small datasets (e.g., Wisz et al.^67^). Applying Maxent in *ENMeval* permits splitting occurrence data into ‘spatial blocks’, which among other benefits decreases the chance of high spatial autocorrelation between training and testing sites, and thereby counters model overfitting^68^. *ENMeval* also includes model selection procedures^69^.

The ENM pipeline consisted of the following steps. Occurrence- and background data were first divided into spatial blocks to decrease chances for high spatial autocorrelation between training and testing sites. Then, a set of initial models was created for these subsets, each with different combinations of parameter settings, which together represent all combinations of regularization multiplier (1–5) and feature class settings [‘linear’ (L), ‘linear & quadratic’ (LC), hinge (H), and linear, quadratic and hinge (LQH)]. The optimal model was defined as the model with the lowest mean percentage of test sites falling outside the predicted range (i.e., lowest mean omission error), and the highest mean evaluation (AUC) value^70,71^. To check whether this model performed significantly better than models based on randomly selected data, a series of 100 null models was created^72^ based on Raes and Ter Steege^21^. Optimal models of *Aedes* *aegypti*, *Aedes* *taeniorhynchus*, *Culex* *quinquefasciatus*, *Culex* *nigripalpus*, *Deinocerites* sp., *Haemagogus* *chrysochlorus* and *Psorophora* *confinnis* s.l. were found to perform significantly better than models based on randomly selected data. See Table 1 for more information on their ecology and habitat preferences. All environmental variables were used in the initial models; no initial correlation assessments were performed. Because Maxent is a machine learning algorithm, (strongly) correlating variables have no effect on performance when predictions are only made for the current time period^73^. However, there is a risk of overparameterisation, and suitability maps based on ecologically relevant variables are more accurate than maps based on arbitrary environmental variables^74–76^. The percentage contribution of each environmental variable was therefore retrieved from the results of the initial models and used to select the most influential variables. If within this selection there were still sets of variables with a correlation of less than − 0.7 or more than 0.7 Pearson’s *r*, the least important variable was removed (based on Jueterbock^77^). The final set of environmental variables was used to generate a new set of models, again for all different combinations of parameter settings. The optimal model from this series was selected as the final model. From this model, plots of the occurrence data divided into spatial blocks, metadata, species-environment relationship plots, and the spatial predictions (suitability maps) were exported as separate files. Taking into account the growing criticism, the evaluation of model performance continues to predominantly rely on the validation of Area Under the Curve (AUC) scores^78,79^. In the context of our investigation, we adopt a nuanced approach to interpreting AUC values: an AUC value of ≤ 0.5 is considered not performing better than random. Those within the range of AUC > 0.5 but < 0.7 are classified as demonstrating poor to moderate performance; AUC values > 0.7 but < 0.9 signify models exhibiting moderate to high performance; and notably, an AUC > 0.9 is indicative of excellent performance^80–82^. On top of AUC values we also evaluated model performance based on the Boyce index^83^. The index ranges from − 1 to 1. A value closer to 1 indicates model predictions align with presences’ distribution while a value closer to − 1 indicates the model predictions do not align with the presence’s distribution. Values around zero indicate the model does not differ from a random model. Variable response curves were also evaluated to understand the correlations between variables and predicted distributions (Figure S2). The entire pipeline can be found under Data Availability Statement.

### Supplementary Information

Supplementary Information.

## Data Availability

All data, including used scripts, can be found on our GitHub repository, link: https://github.com/wouterbeukema/Wouters_et_al_MosquitosBonaire.
