# Supplementary material for: Local environmental factors drive distributions of ecologically-contrasting mosquito species (Diptera: Culicidae)
Source: Sci Rep. 2024 Aug 20;14:19315. doi: 10.1038/s41598-024-64948-y (PMC11336062; doi:10.1038/s41598-024-64948-y)
Supplement: Supplementary file 1 — Supplementary Information. [file 41598_2024_64948_MOESM1_ESM.pdf]

Supplementary Material - Wouters *et al.* *"Local environmental factors drive distributions of ecologically-contrasting mosquito species (Diptera: Culicidae)"*

A. *Aedes aegypti*

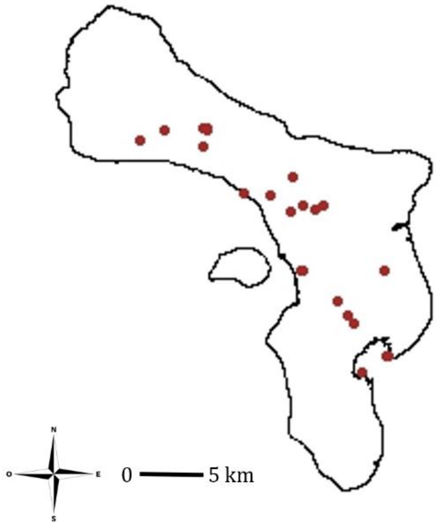

B. *Culex quinquefasciatus*

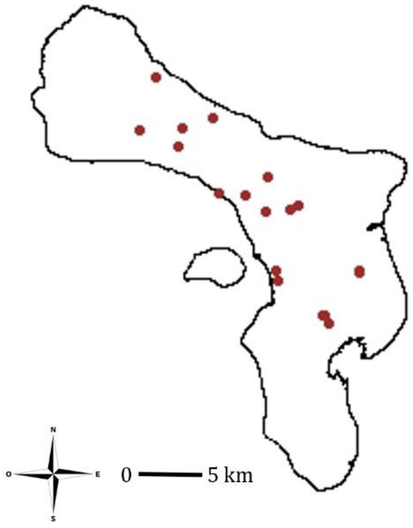

C. *Aedes taeniorhynchus*

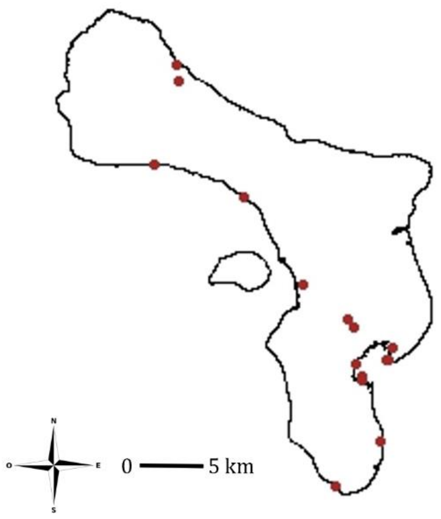

D. *Culex nigripalpus*

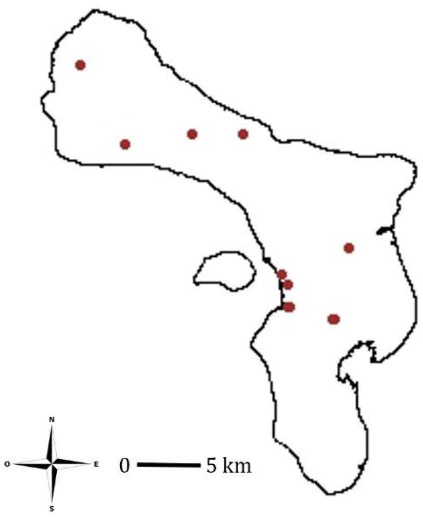

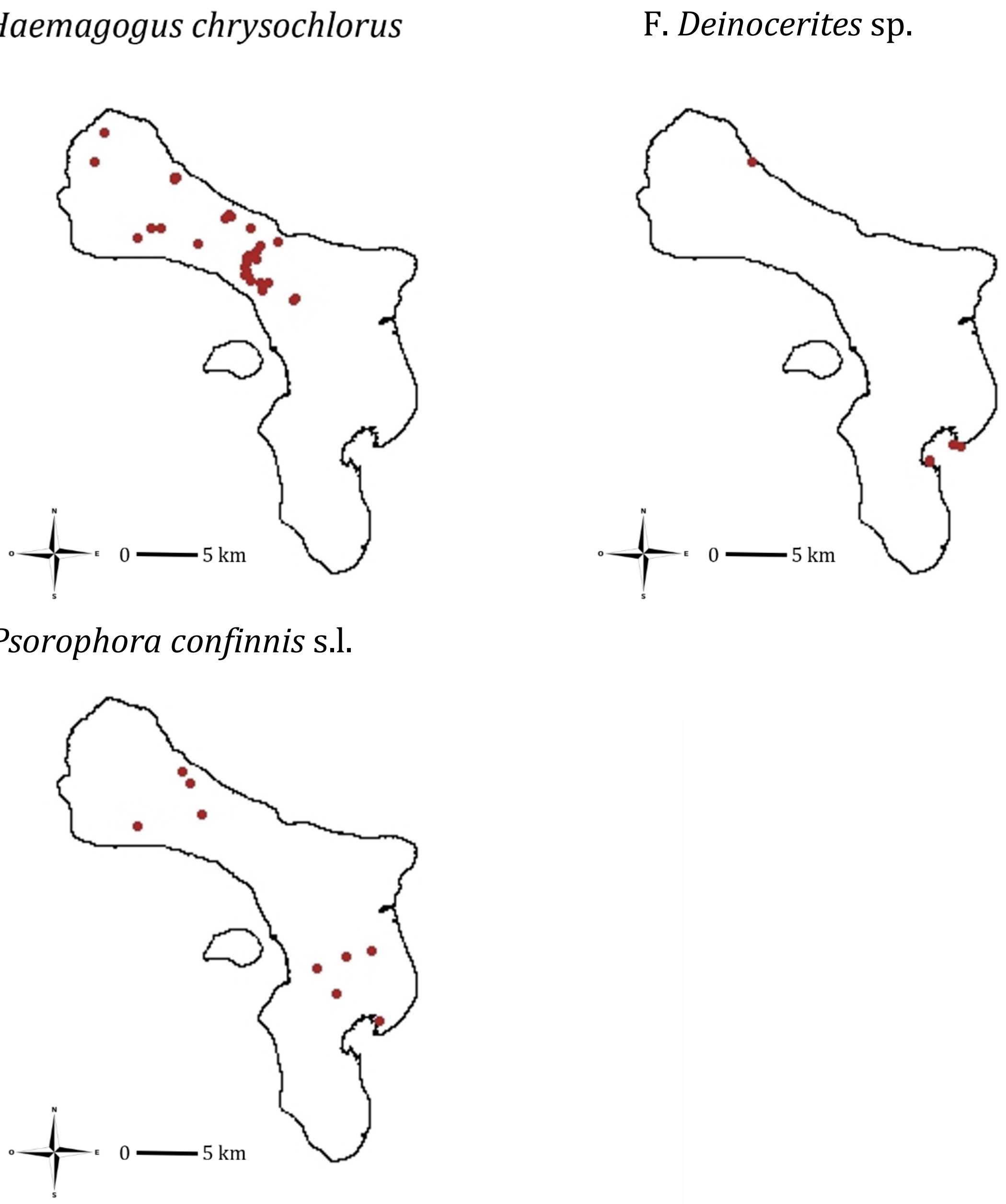

Figure S1. Occurrence maps for (A) *Aedes aegypti* (n=19), (B) *Aedes taeniorhynchus* (n=13), (C) *Culex quinquefasciatus* (n=16), (D) *Culex nigripalpus* (n=10), (E) *Haemagogus chrysochlorus* (n=23), (F) *Deinocerites* sp. (n=4) and (G) *Psorophora confinnis* s.l. (n=9) on Bonaire. Each brown dot represents a positive sampled location (presence) for that specific species. Both larval, pupal and adult stages are incorporated and a single dot might represent more than one individual. Note that the island of Klein Bonaire was not sampled.

Table S1. Mosquito species collected on the island of Bonaire in our field study from 30 November – 14 December 2022.

	Species
	Aedes (Ochlerotatus) taeniorhynchus (Wiedemann, 1821)
	Aedes (Ochlerotatus) scapularis (Rondani, 1848)
	Aedes (Stegomyia) aegypti (Linnaeus, 1762)
	Anopheles (Nyssorhynchus) aquasalis Curry, 1932.
	Culex (Culex) saltanensis Dyar, 1928
	Culex (Culex) nigripalpus Theobald, 1901
	Culex (Culex) quinquefasciatus Say, 1823
	Culex (Melanoconion) spp. (Dyar & Knab, 1906)
	Deinocerites sp.
	Haemagogus (Haemagogus) chrysochlorus Arnell, 1973
	Psorophora (Grabhamia) confinnis s.l. (Lynch Arribálzaga, 1891)
	Uranotaenia (Uranotaenia) lowii Theobald, 1901

Table S2. Model selection and performance per species. Area under the receiver-operating characteristic curve (AUC) values indicates model performance (>0.6 poor performance, 0.6-0.7 moderate performance and <0.7 good performance). Feature class (FC) shows the transformation of the predicted variables, FC can be (a combination of): linear (L), quadratic (Q), hinge (H). The selected regularisation multiplier (RM) can be 0 – 5[1].

Species	AUC value	Boyce index	FC optimal	RM
Aedes aegypti	0.65	0.41	H	5
Aedes taeniorhynchus	0.89	0.31	LQ	2
Culex quinquefasciatus	0.64	0.74	H	5
Culex nigripalpus	0.50	0.47	H	5
Haemagogus chrysochlorus	0.56	-0.11	LQ	2
Deinocerites sp.	0.78	0.63	H	5
Psorophora confinnis s.l.	0.56	0.11	L	5

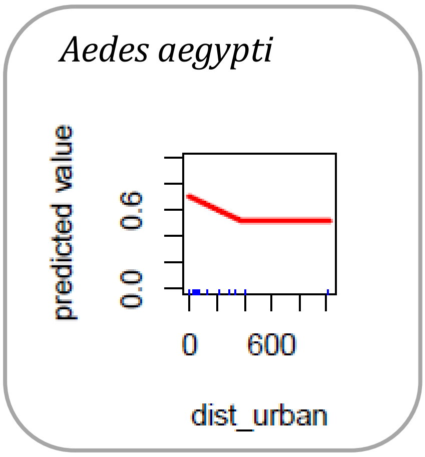

Figure S2 Variable response curve of *Aedes aegypti* indicates a negative correlation with the predicted value of habitat suitability (x axis) and distance to urbanisation (y axis).

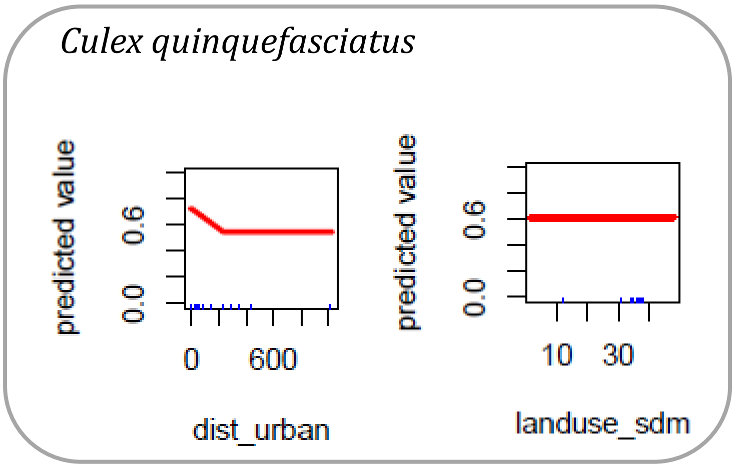

Figure S3 Variable response curves of *Culex quinquefasciatus* indicate i) a negative correlation with the predicted value of habitat suitability (x axis) and ii) distance to urbanisation (y axis) and ii) a correlation with the predicted value of habitat suitability and landuse: mangrove habitat (y-axis, categorical)

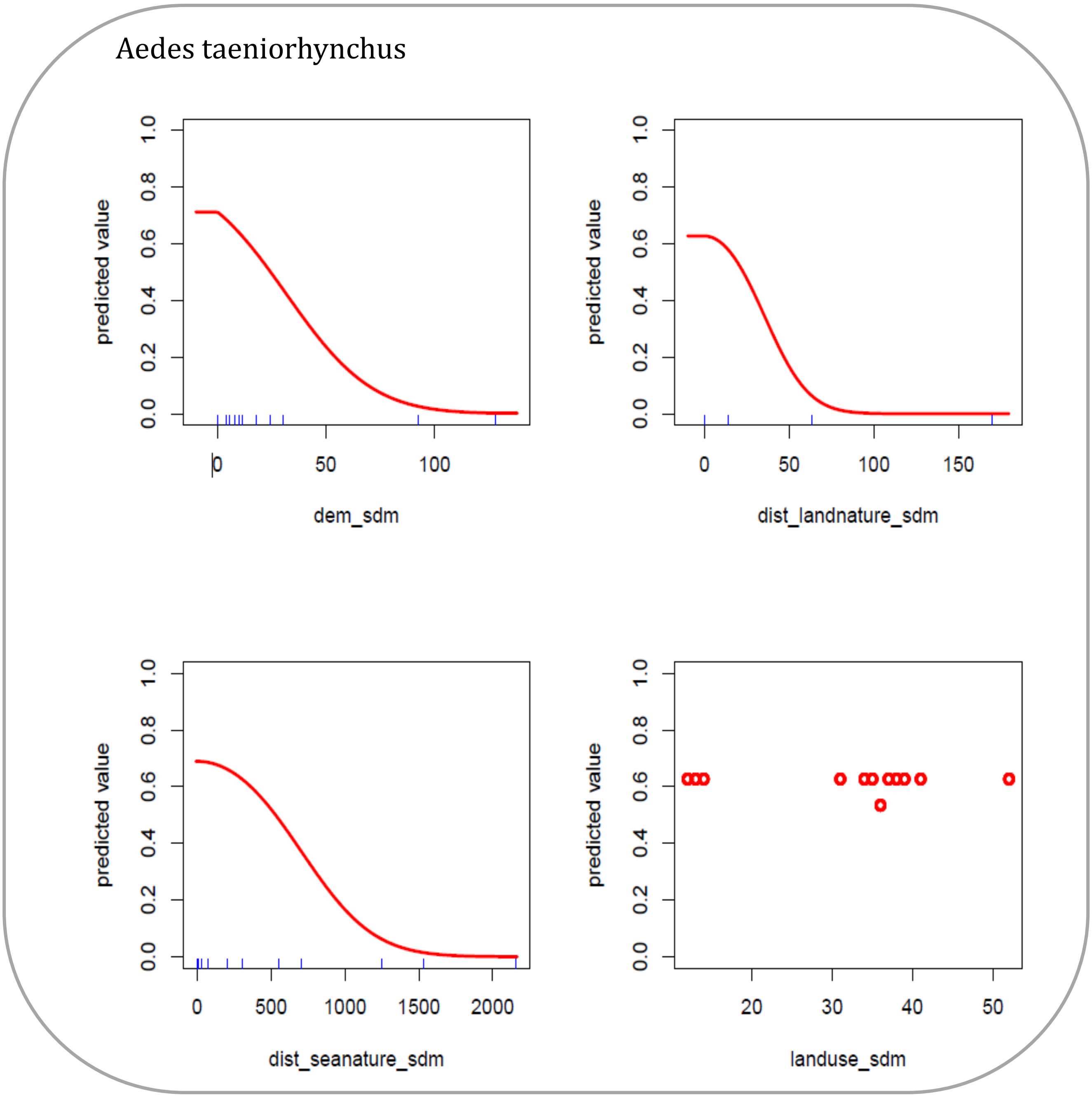

Figure S4 Variable response curves of *Aedes taeniorhynchus* indicate a negative correlation with the predicted value of habitat suitability (x axis) and i) elevation (y axis), ii) distance to natural habitat (y axis), iii) distance to mangrove habitat (y axis) and iv) a correlation with the predicated value of habitat suitability and landuse: mangrove habitat (y axis, categorical).

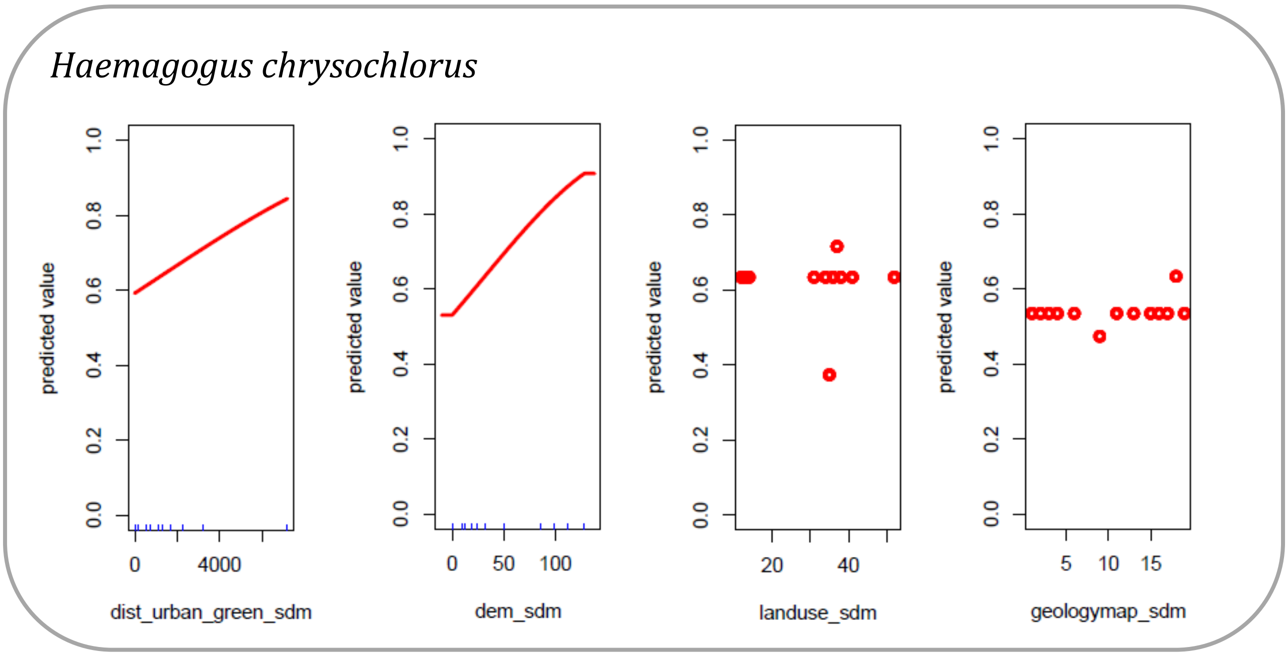

Figure S5 Variable response curves of *Haemagogus chrysochlorus* indicate a positive correlation with the predicted value of habitat suitability (x axis) and i) distance to natural habitat (y axis) and ii) elevation (y axis), iii) a correlation with the predicated value of habitat suitability and landuse: forest and high scrub habitat (y axis, categorical) and iv) geology: high terrace (y axis, categorical).

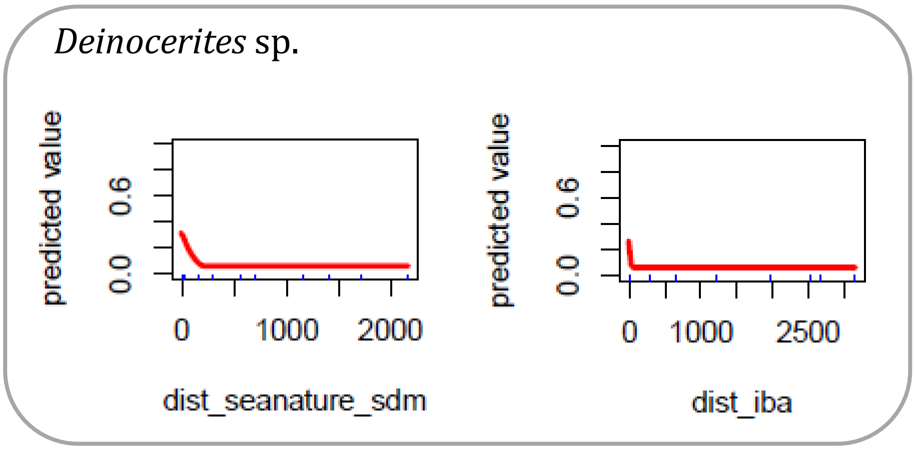

Figure S6 Variable response curves of *Deinocerites* sp. indicate a negative correlation with the predicted value of habitat suitability (x axis) and i) distance to mangrove habitat (y axis) and ii) distance to protected nature area (y axis).

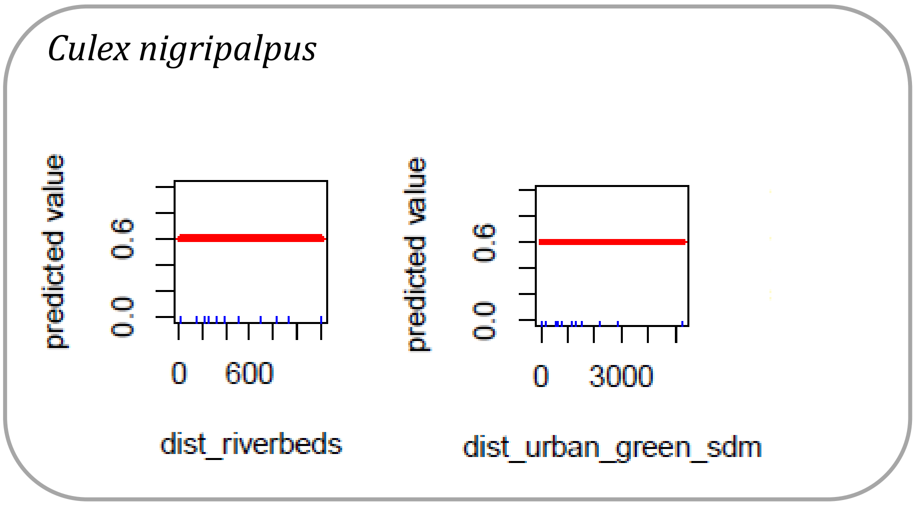

Figure S7 Variable response curves of *Culex nigripalpus* indicate no clear correlation with the predicted value of habitat suitability (x axis) and i) distance to temporary waterbody (y axis) and ii) distance to natural habitat (y axis).

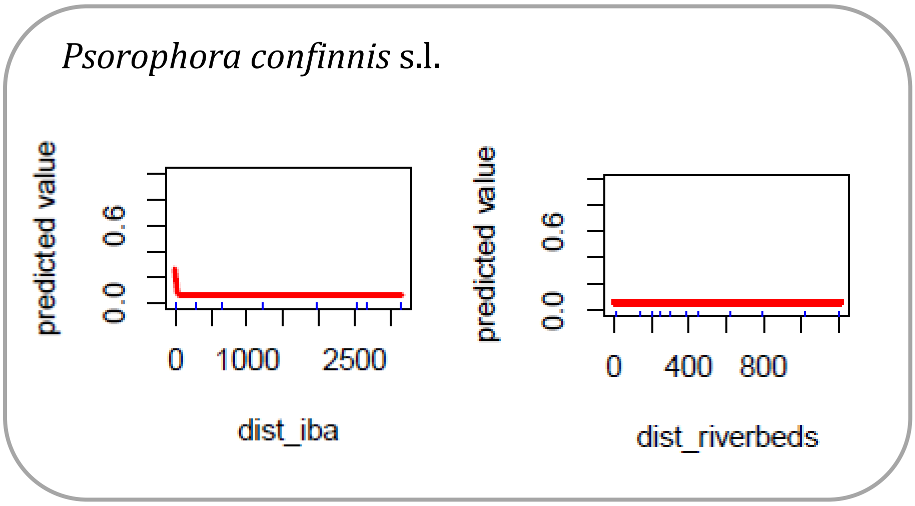

Figure S8 Variable response curves of *Psorophora confinnis* indicate a negative correlation with the predicted value of habitat suitability (x axis) and i) distance protected nature area (y axis) and ii) no clear correlation with distance to temporary waterbody (y axis).

Table S3. Overview of all baseline maps with subcategories that were used to render spatial layers and create models. References are included and all data can be downloaded from the Dutch Caribbean Biodiversity Database (https://www.dcbd.nl/).

Spatial layer with subcategories
Land use[2]
Built-up (urban)
Urban bare soil (urban)
Road (urban)
Urban green
Bare soil and pioneer vegetation (nature)
Sandy beaches (nature)
Low scrub and mangrove (nature)
Low scrub (nature)
Low scrub with cactus (nature)
High scrub (nature)
High scrub with cactus (nature)
Forest
Mangrove
Salina
Salt ponds
Crystalizer ponds
Deep sea (excluded from model input)
Shallow coastal waters
Lagoon
Shallow inland waters
Geology[3]
Eolianite
Lower terrace
Middle terrace
Higher terrace
Seroe Domi formation
Upper Eocene
Intrusive Porphyritic Quartz Diorite
Soebi Bianco congolomerate
Rincon formation
Washikemba formation – mainly Diabases and Tuffs
Washikemba formation – Porphyrites and Tuffs
Washikemba formation – Diabese intrusions
Salinas and other flats
Calcareous sand and dunesand
Coral shingle, beachrock and rampants
Man-made constructions
Salt plains
Mangrove
Water
Soil[4]
Soils of limestone formation
Reddish soils of lower depositional terrace
Reddish soils of middle depositional terrace

	Spatial layer with subcategories
	Yellowish brown soils of middle depositional terrace
	Rock land of depositional and erosional terraces
	Plateau land
	Miscellaneous landtypes of limestone formations
	Soils of the Washikemba formation
	Soils of the plains
	Stony land
	Stony plateau land
	Hilly land
	Alluvial and colluvial soils
	Rooibottom and plain soils
	Soils of the roois
	Soils of the fans
	Other
	Vegetation[5]
	Rhizophora – Batis type
	Sesuvium – Lithophila type
	Conocarpus type
	Lithophila – Euphorbia type
	Strumpfia type
	Euphorbia – Sporobolus type
	Lantana – Capraria type
	Eragrostis – Melocactus type
	Coccoloba – Metopium type
	Haematoxylon – Antirhea type
	Aristida – Jatropha type
	Acacia – Caesalpinia type
	Cordia – Melochia type
	Croton – Haematoxylon type
	Eragrostis – Cyperus type
	Prosopis – Opuntia type
	Casearia – Prosopis type
	Casearia – Bourreria type
	Important Bird and Biodiversity Areas[6]
	Urban areas[7]
	Neighbourhoods[8]
	Antriol
	Belnem
	Hato
	Nikiboko
	Noord Salina
	Playa
	Rincon
	Terkora
	Riverbeds[9]
	Foot paths[10]
	Digital Elevation Model[11]
	Catchment and Waterflows: temporary waterbodies[12]

Supplementary references

1. Kass, J. M. *et al.* ENMeval 2.0: Redesigned for customizable and reproducible modeling of species' niches and distributions. *Methods Ecol. Evol.* **12**, 1602–1608 (2021).
2. Mücher, C. A. . & Verweij, P. J. F. M. *Land Cover Classification Bonaire*. (2020).
3. Koomen, A., van Dorland, G. & Makaske, B. *Geological Map of Bonaire*. (2012).
4. Koomen, A., van Dorland, G. & Makaske, B. *Soil map of Bonaire*. (2012).
5. Debrot, A. O. & Dijkman, E. *Vegetation map of Bonaire*. (1996).
6. Geelhoed, S. C. V. *et al*. *Important Bird and Biodiversity Areas (IBA) map of Bonaire*. (2013).
7. van den Broek, P. *Urban areas map of Bonaire*. (2014).
8. Hennen, W. *Neighbourhoods Bonaire*. (2020).
9. van den Broek, P. *Riverbeds map of Bonaire*. (1996).
10. van den Broek, P. *Foot path map of Bonaire*. (1996).
11. North American Space Agency. *Digital Elevation Model of Bonaire*. (2009).
12. Mücher, C. A. *Catchments and waterflows of Bonaire*. (2019).
